# It probably worked: a Bayesian approach to evaluating the introduction of activity-based hospital payment in Israel

**DOI:** 10.1186/s13584-022-00515-y

**Published:** 2022-02-15

**Authors:** Ruth Waitzberg, Martin Siegel, Wilm Quentin, Reinhard Busse, Dan Greenberg

**Affiliations:** 1 The Smokler Center for Health Policy Research, Myers-JDC-Brookdale Institute, Jerusalem, Israel; 2 Department of Health Policy and Management, School of Public Health, Faculty of Health Sciences, Ben-Gurion University of the Negev, Be’er Sheva, Israel; 3 Department of Health Care Management, Faculty of Economics & Management, Technische Universität Berlin, Straße des 17. Juni 135, 10623 Berlin, Germany; 4 Department of Empirical Health Economics, Technische Universität Berlin, Straße des 17. Juni 135, 10623 Berlin, Germany; 5 European Observatory on Health Systems and Policies, Brussels, Belgium

**Keywords:** Hospital payment, Economic incentives, Payment reform, Procedure-related group payments, Bayesian estimation, Israel

## Abstract

**Background:**

In 2013–2014, Israel accelerated adoption of activity-based payments to hospitals. While the effects of such payments on patient length of stay (LoS) have been examined in several countries, there have been few analyses of incentive effects in the Israeli context of capped reimbursements and stretched resources.

**Methods:**

We examined administrative data from the Israel Ministry of Health for 14 procedures from 2005 to 2016 in all not-for-profit hospitals (97% of the acute care beds). Survival analyses using a Weibull distribution allowed us to examine the non-negative and right-skewed data. We opted for a Bayesian approach to estimate relative change in LoS.

**Results:**

LoS declined in 7 of 14 procedures analyzed, notably, in 6 out of 7 urological procedures. In these procedures, reduction in LoS ranged between 11% and 20%. The estimation results for the control variables are mixed and do not indicate a clear pattern of association with LoS.

**Conclusions:**

The decrease in LoS freed resources to treat other patients, which may have resulted in reduced waiting times. It may have been more feasible to reduce LoS for urological procedures since these had relatively long LoS. Policymakers should pay attention to the effects of decreases in LoS on quality of care. Stretched hospital resources, capped reimbursements, retrospective subsidies and underpriced procedures may have limited hospitals' ability to reduce LoS for other procedures where no decrease occurred (e.g., general surgery).

**Supplementary Information:**

The online version contains supplementary material available at 10.1186/s13584-022-00515-y.

## Background

Means of payment affect healthcare provider decision-making, including patient admission policies and treatment decisions [1, 2]. Payments to health providers are therefore used by policymakers to achieve health system objectives. Typically, policymakers pursue multiple objectives, such as increasing technical efficiency (reducing costs without undermining quality or increase productivity without increasing costs but maintaining quality), incentivizing providers to admit and treat patients based on need, and providing high quality care, while controlling expenditures. Since 1980s, the United States and other high-income countries shifted to activity-based payments such as DRGs as hospital payment mechanisms. The aim was to achieve a more appropriate and equitable allocation of resources and increase transparency by better measurement of activity, once payment was tied to activity [3]. DRGs incentivize increasing the number of cases, reducing the cost per patient, and reducing (avoidable) costs. Hospitals can reduce costs per patient by reducing the number of services provided to each, reducing length of stay (LoS), or selecting patients with low risk and low comorbidity. According to the literature, it is unclear how activity-based payments affect quality of care [3–5].

Evidence on the effects of DRGs on healthcare providers’ behavior as measured by LoS and volume of patients has been mixed, with different results in different countries [6–10]. In certain countries DRGs led to reduced LoS or increased volume of patients while in others the impact on LoS was not significant, suggesting that the effects differ as a function of context. For example, in England, DRGs led to increased volume of care, decreased LoS [11, 12] and service distortion, i.e., not providing the most appropriate care, under- or overprovision [13]. In Poland, the introduction of DRGs also led to service distortion [14]. In Austria, LoS decreased during the first 10 years (1997–2007) and patient turnover increased in the first 20 years, but decreased again after creation of new outpatient care codes [15]. Despite that the local variants of DRGs in these three countries groups patients primarily based on the main procedure [17], these mixed effects point to the importance of examining the impact of payment reform where and how it occurs. The present study evaluated whether the Israeli local variant of DRGs, the Procedure related group (PRGs), that also groups patients by procedure, caused similar effects as in England, Austria and Poland, or whether local context led to different outcomes.

### The Israeli hospital market and the PRG payment reform

Universal health coverage in Israel is based on National Health Insurance (NHI), in which four competing health plans (HPs) are responsible for purchasing healthcare to all residents. Hospital revenue comes primarily from the sale of services (90% in 2017), of which 84% is paid by health plans (HPs)[16]. The methods of payment for different hospital services and its tariffs are set by the MoH, covering variable and fixed costs. About one third of hospital revenue comes from PRGs payments, a local variant of DRGs similar to hospital payments in England, Austria, and Poland [17]. Whereas DRGs cluster patients according to diagnoses requiring similar resources and costs, PRGs group patients receiving similar medical procedures, resources and costs. PRG-based payments in Israel are not adjusted for case mix or illness severity. The remaining inpatient care is paid by per-diem payments (PDs). In October 2019, there were 12 PD tariffs according to ward type and LoS (e.g., higher for the first three than subsequent days), and about 360 PRG codes [18].

One peculiarity of Israel’s hospital payment system is that there are two major constraints on hospital revenues. The first is an annual cap on payments by each HP. If payments exceed the cap, hospitals receive only a fraction of payment for the difference. Further constraints are imposed by alternative reimbursement contracts negotiated between HPs and hospitals [19], representing about 21% of hospitals’ gross income [16]. Reduced income may limit the effectiveness of payments’ incentives to increase activity if hospitals do not receive full payment beyond the cap or agreements. In addition, incentives to reduce costs per case are high, since marginal revenues are less likely to cover marginal costs.

PRG payments in Israel were defined for 30 common procedures in the 1990’s. In 2010, the MoH advocated for a broader adoption of PRGs in place of PDs. Payment reform was implemented in three waves over several years, each for different clinical domains. The first between 2010 and 2012 created about 80 new PRG codes, mostly for orthopedic urgent care procedures. The second wave was between 2013 to 2014, when the MoH created 65 PRG codes to pay for procedures mainly in urology, general surgery, ophthalmology, head and neck surgery. The last wave in 2015 covered 50 elective orthopedic procedures.

The PRG-payment expansion was intended to enhance transparency by precise activity coding and improved distribution of funds using a refined unit of payment method. The intent was to reduce waiting times [20], to increase patient turnover and to shorten LoS [3, 21]. Yet because PRGs are not adjusted for case-mix or illness severity, hospitals are incentivized to provide overpriced procedures and select profitable, low intensity patients over those likely to require more resources [22]. For this study, we assessed whether and how the second wave of the of expansion of PRG-based payments in 2013 and 2014 achieved one of its objectives; namely to shorten LoS. We analyzed these changes for 14 different procedures.

Initial research in Israel examined changes in patient turnover and LoS for certain procedures after PRGs were first introduced in the 1990’s [23] and after adjustments to PRG payments, for faster treatment of hip replacement [24]. These studies examined five major procedures: cholecystectomy, hysterectomy, hip replacement, cataract surgery and treatment for acute myocardial infraction (AMI), consistent with previous similar payment reforms assessment [7, 12, 13, 25–27].

Waitzberg and colleagues [28] examined the recent expansion of PRG-based payments and reported no statistically significant associations of the payment reform with patient turnover or LoS (aggregated) at the ward level. However, examination of change at the ward level may not be sufficiently sensitive. This is because in each ward, there are different types and levels of payment for different procedures, and the shifts in similar procedures to achieve higher marginal profits may not be visible in aggregate data. Second, increases in LoS in one procedure may be offset by decreases in another.

To address this potential limitation, we examined the impact of PRG-based payments on average LoS at the procedure level. Based on previous findings, we expected payment change to lower costs per case by reducing LoS. We also examine change in 14 procedures less frequently examined in the literature. By expanding types of procedures, we may pinpoint how clinical fields respond differently to payment reform. And though the effects of DRG-like payments have been widely studied in other contexts with inconsistent findings across countries, Israel’s unique hospital market allows for the analysis of the potential effects of economic incentives on hospitals with capped reimbursements, stretched resources, and underpriced procedures (due to discounts from health plans beyond the MoH’s price list). We take a different approach than previous research by applying survival analyses to LoS data, where the “event” is the discharge from the hospital instead of death as common in survival analysis in medical research, and the duration is the LoS instead of the survival time. In this approach, the effect of the reform is modeled as the relative change in average LoS when comparing the periods before and after the reform. We control for age, gender, comorbidity and hospital ownership and location. Technically, this approach is more suitable than for example linear models because LoS is often right-skewed and cannot be negative ($\ge 0$), and would thus violate the assumption of normally distributed error terms required in most linear models (see Box for a summary of the added value of this study in terms of methods and findings).

**Table:** 

**Box: the added value of this study**
Many studies assessed the effects of adoption of activity-based payments to hospitals on outcome measures such as lengths of stay (LoS), volumes of care and quality. Most studies apply a differences-in-differences approach. Findings are mixed, but in many countries LoS decreased after a payment reform
**What this study adds**
We analyzed these effects on 14 procedures less explored in the literature
We applied a survival analysis, which allowed us to examine strictly non-negative and right-skewed data and opted for a Bayesian approach to estimate relative change in LoS
In Israel, hospitals reduced LoS for half of the procedures examined, mostly urological procedures. LoS reduction ranged between 11% and 20%
Measuring relative change in LoS allowed us to examine various patterns of LoS across procedures
The stronger effects were seen in patients with long stays

## Materials and methods

### Data and measurement

To analyze the impacts of the second wave of the PRG reform, when many PRG codes were created in July 2013 and January 2014, we used administrative data from the MoH for selected procedures performed between 2005 and 2016 in all non-profit hospitals (97% of the acute care beds). Of the 65 PRG codes created in this period, we sampled 14 procedures (listed in Table 1) which met the following inclusion criteria:

1. Procedures for which PRG payment codes were first created between July 2013 and January 2014, to capture only those that belong to the period of the second ‘wave’.
2. Procedures with at least 200 cases per year, to capture relatively high-volume procedures, that have a relatively high impact on hospitals’ activity and income.
3. Procedures with PRG codes that have not been changed or regrouped since their inception, otherwise it would be difficult to isolate the effects of the change in payment method from effects of change in pricing (procedures for which a PRG code existed but changed because they were grouped with other procedures, might have different prices after the change). In addition, the MoH was not able to distill data when the grouping of ICD-9 codes changed by PRG code.
4. Procedures performed in inpatient settings only, as hospitals do not report ICD-9 and PRG codes for procedures performed in outpatient settings and the MoH could not distill data from this setting
5. Elective procedures only, because hospitals can choose the type of payment (PRG or PD) for urgent care. When a procedure is underpriced, and has long stays, the hospital has incentives to charge PD instead of PRG, and the effects of the reform are not seen.
6. No trauma procedures, since multiple procedures are often performed, and hospitals only report the main procedure. In these cases, some procedures might not be properly charged, and again the economic incentives of the reform are distorted.

**Table 1:** List of procedures

	Abbreviated procedure	PRG procedure	Clinical field	PRG code created
1	Open abdom. hernia rep	Open anterior abdominal wall hernia repair, excluding post-operative ventral hernia	General surgery	1/1/2014
2	Lap. abdom. hernia rep	Laparoscopic anterior abdominal wall hernia repair, excluding post-operative ventral hernia	General surgery	1/1/2014
3	Lap. diaphragm. hernia rep	Laparoscopic diaphragmatic hernia repair	General surgery	1/1/2014
4	Anti-reflux surgery	Esophagogastric sphincteric competence creation	General surgery	1/1/2014
5	Open splenect	Open partial or complete splenectomy	General surgery	1/1/2014
6	Perct. nephrostomy	Percutaneous nephrostomy, including fragmentation	Urology	7/1/2013
7	Open part. nephrect	Open partial nephrectomy	Urology	7/1/2013
8	Lap. part. nephrect	Laparoscopic partial nephrectomy	Urology	7/1/2013
9	Open complete nephrect	Open complete nephrectomy, including ureterectomy	Urology	7/1/2013
10	Lap. complete nephrect	Laparoscopic complete nephrectomy, including ureterectomy	Urology	7/1/2013
11	Diag. ureterosc	Diagnostic ureteroscopy with or without biopsy	Urology	7/1/2013
12	Therap. ureterosc	Therapeutic ureteroscopy including retrograde intrarenal surgery	Urology	7/1/2013
13	Lap. salpingect	Laparoscopic unilateral or bilateral salpingectomy	Gynecology	7/1/2013
14	Lap. salp.-oophorect	Laparoscopic unilateral or bilateral salpingo-oophorectomy	Gynecology	7/1/2013

Procedures belonged to general surgery (5 procedures), urology (7) and gynecology (2), and were selected and pooled based on the ICD9 codes listed in Table A1 in the Additional file 1: Appendix. Our dataset comprised information on all inpatient cases where patients underwent one of the selected procedures listed in Table 1. Due to privacy regulations, aggregate patient data were reported by age, sex, hospital, year and procedure. Inflating the data using the group sizes as frequency weights allowed us to identify the age-group and sex, procedure performed, and hospital for each patient. However, only group medians for the Charlson comorbidity index and the group means for the LoS were available for the analysis, which may lead to underestimation of variance for these two variables.

### Data preparation

We consider the top 5% of reported LoS as outliers and decided to drop them because they likely represent the most severe, complicated cases, where medical considerations usually takes precedence over cost. Including these cases could obscure tradeoffs between economic and medical considerations. Computing the 95% quantiles for LoS separately for each procedure yielded that LoS was up to the 26-fold of the respective 95% quantiles for some procedures. Including such severe outliers could distort the algorithm and bias estimation results even when assuming a distribution which is designed for right-skewed data.^1^ Summary statistics for the complete sample including outliers appear in Tables A2 and A3 in the Additional file 1: Appendix.

### Survival analyses

To estimate the effects of the payment reform on LoS, we performed survival analyses using a Weibull distribution, where we model the scale parameter $\lambda$ using a linear mixed effect containing both fixed and random effects. The Weibull distribution is used in survival analyses to account for right-skewed distributions of non-negative waiting or survival times and showed the best fit when compared to the observed distribution of LoS in our data. The estimated parameters can be interpreted as relative changes in the expected LoS, which warrants a better comparability of the results across procedures than absolute changes because the magnitude of absolute changes in LoS will depend on the average LoS in a procedure. We assumed that LoS $\tau$ followed a Weibull distribution,

$$\tau_{it} \sim {\text{Weibull}}\left( {\lambda_{it} ,p} \right),$$

where $\lambda_{it}$, and thus the survival function and expected length of stay $E{[}\tau_{it} {|}x_{it} ,\lambda_{it} ,p]$ for an individual $i$ in a year $t$, depend on a set of $k$ exogenous regressors $x_{k,it}$:

$$\lambda_{it} = \beta_{0} + \mathop \sum \limits_{k} \beta_{k} x_{k,it} + u_{t} + \epsilon_{it} .$$

In this specification, the $$\beta s$$ (age, sex, comorbidity and different hospital characteristics) are fixed effects because they are constant across individuals and years. In other words, the $$\beta s$$ were assumed to have the same relative effect on a patient’s LoS each year. Random effects ($$u_{t}$$) allow for a basic LoS which varies across years to accommodate unobserved year-specific heterogeneity (e.g., technical progress, 2011 physicians’ strike, introduction of other PRG codes for procedures not included in our data or changes in the supply or demand of healthcare). The shape parameter $$p$$ accounts for potential change in individual likelihood of release from the hospital over LoS. In technical terms, it enables the hazard function $$h\left( {\tau_{it} ;\lambda_{it} ,p} \right) = p\lambda_{it} \tau_{it}^{p - 1}$$ to increase (if $$p > 1$$) or to decrease (if $$p < 1$$) with $$\tau$$. The hazard function does not vary with $$\tau$$ and the Weibull-distribution simplifies to an exponential distribution if $$p = 1$$. This specification also implies that the effects $$\beta_{k}$$ of the explanatory variables on the hazard function increase (decrease) with $$\tau$$ if $$p > 1$$ ($$p < 1)$$. The shape parameter allows us to distinguish two potential scenarios: First, immediate discharge after admission to hospital is improbable, but becomes increasingly likely the longer the stay ($$p > 1)$$. Second, decreasing probability of discharge is likely if it is assumed that most patients will be released sooner than later; only more severe cases with complications require extended stays. This corresponds to a lower likelihood of release the longer the elapsed hospital stay $$(p < 1)$$. See [29]for an extensive discussion of survival analyses.

### Bayesian estimation

We opted for a Monte-Carlo-Markov-Chain (MCMC) based Bayesian analysis using the Metropolis–Hastings-algorithm, because data were grouped by age and sex, and comprised summary statistics (mean LoS and median Charlson index). This means that observed variance for explanatory and outcome variables within the groups defined by age, sex, hospital and year is zero, and estimates of overall variance for explanatory and outcome variables may be attenuated. Estimation approaches in which standard errors derived from the variance of the error term would thus likely yield unreliable confidence intervals and potentially biased results.

The most important difference between the frequentist and Bayesian approach used here is that the latter considers estimated parameters as random variables drawn from a distribution to be estimated. Therefore, our estimation aimed to describe the distributions of parameters reflecting observed patterns in the data. These distributions are used to derive expected parameter values and the probability that a parameter lies within a certain interval. In the frequentist approach, uncertainty is mainly the result of sample variations, where the variance of a parameter is the outcome of randomness in the sampling process, such that different samples yield different results, but the perfect (infinite) sample would find the true parameter with certainty. The frequentist approach thus considers parameters as fixed but unknown, and assumes that confidence intervals include the fixed but unknown parameter with a certain probability.

As priors, we use fairly, but not fully, uninformative distributions. We drew fixed effect parameters in the MCMC simulation based on a normal distribution with mean = 0 and standard deviation $= 100$, such that draws closer to zero were more likely, but anything in the $\left( { - \infty ;\infty } \right)$-interval was possible (consistent with null hypotheses $\beta_{0} = 0$ and $\beta_{k} = 0 \forall k$, and $\ln \left( p \right) = 0$). Variance for year-fixed effects $u_{t}$, $\sigma_{{u_{t} }}^{2}$ was estimated as an Inverse Gamma distribution with a parameter of $\alpha = \beta = 0.01$ as the prior distribution, which allowed us to draw from a $\left( {0,\infty } \right)$ interval during the MCMC simulation. Because of our fairly complex design, we allowed the survival analysis algorithm to run for a burn-in period of 15 million simulations to guarantee convergence before recording 100,000 MCMC simulations. Computations were performed with Stata 15.1.

## Results

### Descriptive statistics

Table 2 reports the descriptive statistics for all inpatient cases included in our analyses. Case numbers varied widely across procedures, ranging from 1161 patients who underwent laparoscopic complete nephrectomy to 21,517 who had a therapeutic ureteroscopy. Median age for most procedures was between 55 and 64 years. The youngest patients underwent gynecological laparoscopic salpingectomy whereas urology patients were comparatively old. The proportion of women was lowest for urology-related procedures, and somewhat higher for most surgeries. The median Charlson index of 0 suggests no comorbidities for most patients who underwent gynecology-related procedures and most surgery-related procedures. By contrast, those who underwent urological treatments had comparatively more comorbid conditions with median Charlson indices of 2 across all types of nephrectomies.

**Table 2:** Sample characteristics and number of patients

Procedure	Overall				Before reform (2005–2013)			After reform (2014–2016)		
	N	Median age group	Percent women	Median Charlson index	Median age group	Percent women	Median Charlson index	Median age group	Percent women	Median Charlson index
*General surgery*										
Open abdom. hernia rep	2549	55–64	53.8%	0	55–64	53.9%	0	45–54	53.3%	0
Lap. abdom. hernia rep	5628	45–54	54.7%	0	45–54	55.6%	0	45–54	52.6%	0
Lap. diaphragm. hernia rep	1248	55–64	68.8%	0	55–64	69.1%	0	55–64	68.6%	0
Anti-reflux surgery	2608	35–44	51.9%	0	35–44	51.3%	0	35–44	53.6%	0
Open splenect	4451	55–64	47.7%	1	45–54	46.5%	1	55–64	51.1%	2
*Urology*										
Perct. nephrostomy	5361	45–54	38.8%	0	45–54	39.2%	0	55–64	37.9%	0
Open part. nephrect	2921	55–64	38.4%	2	55–64	39.3%	2	55–64	36.7%	2
Lap. part. nephrect	1245	55–64	37.7%	2	55–64	37.5%	2	55–64	39.0%	2
Open complete nephrect	6832	55–64	42.7%	2	55–64	43.4%	2	55–64	40.9%	2
Lap. complete nephrect	1161	55–64	41.4%	2	55–64	42.2%	2	55–64	38.9%	2
Diag. ureterosc	8133	55–64	30.7%	0	55–64	31.6%	0	55–64	28.8%	0
Therap. ureterosc	21,517	45–54	28.3%	0	45–54	28.6%	0	45–54	28.0%	0
*Gynecology*										
Lap. salpingect	5680	25–34	100%	0	25–34	100%	0	35–44	100%	0
Lap. salp.-oophorect	13,984	45–54	100%	0	45–54	100%	0	55–64	100%	0
Overall	83,318	45–54	52.94%	0	45–54	53.61%	0	45–54	51.74%	0

The descriptive statistics in table two indicate that data before and after the 2013–2014 reform on patient demographics did not change noteworthily. The male/female-ratio was similar before and after the reform. The median age increased for four procedures, but not in the overall sample. The median Charlson index changed only for open splenectomy. In other words, any change in LoS is unlikely to be the result of changes in the demographic profile of the patients over time.

Table 3 reports average LoS for each procedure. Before reform, mean LoS ranged from 2.7 days for laparoscopic salpingectomy to 13.6 days for anti-reflux surgery. LoS decreased for all 14 procedures and was statistically significant for most procedures, except for Laparoscopic abdominal hernia repair, Laparoscopic diaphragmatic hernia repair and anti-reflux surgery.

**Table 3:** Length of stay in days before and after the reform

Procedure	N	Overall		Before reform (2005–2013)		After reform (2014–2016)		p value
		Mean	S.E	Mean	S.E	Mean	S.E	
*General surgery*								
Open abdom. hernia rep	2549	3.021	0.042	3.167	0.076	2.623	0.068	< 0.001
Lap. abdom. hernia rep	5628	2.848	0.027	2.884	0.049	2.759	0.046	0.063
Lap. diaphragm. hernia rep	1248	4.796	0.087	4.937	0.117	4.642	0.106	0.062
Anti-reflux surgery	2608	13.375	0.316	13.463	0.605	13.113	0.548	0.668
Open splenect	4451	13.218	0.127	13.597	0.237	12.190	0.226	< 0.001
*Urology*								
Perct. nephrostomy	5361	6.035	0.030	6.344	0.046	5.312	0.041	< 0.001
Open part. nephrectomy	2921	6.849	0.046	7.143	0.080	6.128	0.072	< 0.001
Lap. part. nephrect	1245	5.920	0.052	6.333	0.064	5.189	0.060	< 0.001
Open complete nephrect	6832	8.322	0.049	8.647	0.090	7.445	0.089	< 0.001
Lap. complete nephrect	1161	6.590	0.074	6.712	0.150	6.273	0.144	0.035
Diag. ureterosc	8133	3.550	0.018	3.764	0.029	3.111	0.026	< 0.001
Therap. ureterosc	21,517	3.368	0.009	3.493	0.013	3.190	0.011	< 0.001
*Gynecology*								
Lap. salpingect	5680	2.665	0.010	2.650	0.017	2.698	0.017	0.045
Lap. salp. -oophorect	13,984	2.935	0.009	2.964	0.014	2.880	0.013	< 0.001
Overall	83,318	4.863	0.017	5.167	0.025	4.264	0.024	< 0.001

Average LoS for the full period and separately for the pre- and post-reform. The p-value column reports the p-value for a two-sided t-test of no difference between pre- and post-reform ($\Delta {\text{LoS}} \ne 0$)

### Estimation results

Table 4 reports the estimated relative changes in LoS with reform calculated by Bayesian mixed-effects survival analyses (see Table A4 in the Additional file 1: Appendix for the full estimation results). Given posterior distributions, most point estimates $E\left[ {tr} \right]$ for time ratios were $< 1$, indicating that LoS was lower after the reform for most procedures, notably in Urology. For example, the estimated ratio for open splenectomy of 0.894 indicates that the expected LoS for patients who underwent this procedure after the reform was 89.4% of the expected LoS before reform; in other words, it decreased by 10.6%. Table 4 further indicates that LoS was reduced by 11–20% for urological procedures after the reform (except laparoscopic complete nephrectomy).

**Table 4:** Change in length of stay

Procedure	Time ratio	Posterior Pr[tr < 1]	HPD-based 95% credibility interval	Effective sample size	Acceptance rate
*General surgery*					
Open abdom. hernia rep	0.867	0.948	(0.718; 1.034)	9260	0.368
Lap. abdom. hernia rep	0.974	0.666	(0.833; 1.118)	11,966	0.360
Lap. diaphragm. hernia rep	1.017	0.412	(0.865; 1.172)	23,134	0.385
Anti-reflux surgery	1.050	0.265	(0.896; 1.216)	35,752	0.351
Open splenect	0.894*	0.968	(0.784; 1.002)	25,060	0.373
*Urology*					
Perct. nephrostomy	0.853**	0.998	(0.769; 0.940)	8050	0.385
Open part. nephrect	0.871*	0.979	(0.756; 0.990)	10,308	0.375
Lap. part. nephrect	0.801*	0.984	(0.640; 0.967)	3241	0.360
Open complete nephrect	0.891*	0.957	(0.767; 1.013)	9207	0.392
Lap. complete nephrect	0.950	0.834	(0.843; 1.061)	24,059	0.363
Diag. ureterosc	0.852**	0.996	(0.759; 0.949)	8251	0.365
Therap. ureterosc	0.892*	0.968	(0.783; 1.002)	1489	0.360
*Gynecology*					
Lap. salpingect	0.957	0.830	(0.864; 1.055)	4082	0.350
Lap. salp.-oophorect	0.929	0.940	(0.840; 1.021)	2774	0.394

*Significant at the 95% level

**Significant at the 99% level (both for one-sided test of [tr < 1]); the 95% HPD interval limits correspond to the 2.5% and 97.5% quantiles of the a-posteriori distributions

Since interpretation of parameter estimates for Bayesian analyses differs from frequentist analyses, we also employed a different approach to hypothesis testing. Analogously to parametric hypothesis-testing, we applied both 95% and 99% certainty thresholds to identify time ratios significantly < 1 (bear in mind that the p-value is the conditional probability that the null hypothesis is true). The third column in Table 4 presents the a posteriori probability that the time ratio would be below 1 after reform (i.e., ceteris paribus, significantly shorter LoS). For example, the conditional probability given all other information in the model for shorter LoS after the reform (Pr[$tr\left\langle 1 \right|x_{it} ]$) is 96.8% for patients undergoing open splenectomy. If this probability exceeds the threshold of 95% (99%), the result is considered statistically significant, as it is the case for open splenectomy. Based on this approach, we found significant decreases of LoS for 7 at 95% (2 at 99%) of the 14 procedures examined. At the 95% threshold, LoS decreased significantly for only one of five general surgery procedures (open splenectomy) but six of seven urological procedures (i.e., all except laparoscopic complete nephrectomy). We found no significant decrease in LoS for either gynecology procedure examined and four of five general surgeries.

**Fig. 1:**
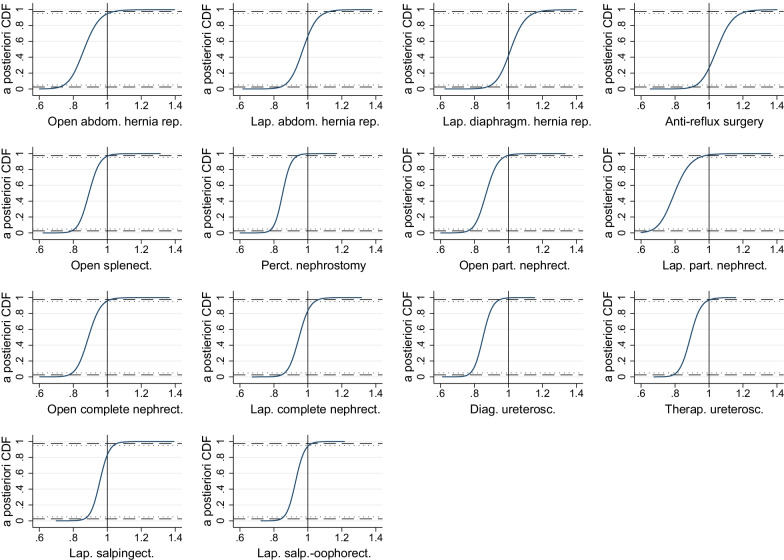
CDF of estimated time ratios (before vs. after reform). A-posteriori cumulative distribution functions (CDFs) of the time ratios. Values on *y*-axis indicate probability to observe the values indicated on the *x*-axis or less: ${\text{Pr}}\left( {tr \le x} \right)$

Figure 1 illustrates the *a-posteriori* cumulative distribution functions (CDF) of the estimated time ratios. For example, the CDF in the plot for open splenectomy is 96.8% for a time ratio of 1, indicating that the probability for decreased LoS is 96.8%. For most procedures, the curves exhibit the steepest increase for time ratios between 0.8 and 1, suggesting high probabilities for a shortened length of stay even for some procedures where changes were not significant. The intersections of the curves with the dashed lines correspond to the 95% credibility intervals reported in Table 4, the intersections with the dotted lines indicate credibility intervals of 90%.

### Controls

Tables A4–A6 in the Additional file 1: Appendix present the complete estimation results for the control variables in all models. The estimation results for the controls are mixed: For example, a higher Charlson comorbidity index is significantly associated with a longer LoS (indicated by the tr > 1) for most procedures, exhibits no significant association with the LoS for laparoscopic hernia repair and laparoscopic partial nephrectomy, and is even associated with a shorter LoS for laparoscopic salpingectomy. The mostly insignificant estimates for age may be explained by the comparatively strong associations of the Charlson comorbidity index with LoS, as the comorbidity status may be strongly correlated with age, but is likely to be even more relevant than age itself. The location of a hospital has mixed associations with the LoS. For both gynecological procedures, two surgical and two urological procedures, the results show significantly positive associations of a location in the periphery with the LoS, whereas for two laparoscopic surgical procedures, a significantly negative association between LoS and peripherical location was observed. Being treated in a hospital that provides tertiary care is significantly associated with a longer LoS in both gynecological procedures, three surgical procedures and five urological procedures. LoS is significantly shorter in tertiary hospitals only for laparoscopic diaphragm repair. Hospital ownership was found to have a significant association with LoS: NGO-ownership is associated with a shorter LoS compared to hospitals publicly owned by the MoH or municipalities for both gynecological procedures, four surgical procedures and two urological procedures. Hospitals owned by Clalit health plan exhibit significantly shorter LoS for gynecological procedures, three surgical procedures and four urological procedures, but a longer LoS for the remaining three urological procedures. The random effect of years contributed minimally to variation in LoS, suggesting that year-specific effects had only a minor influence on LoS (see Table A4 in the Additional file 1: Appendix).

The shape parameter $p$, which indicates whether the likelihood to be released increases or decreases over the already elapsed LoS, is significantly $> 1$ for all procedures (this follows from $\ln \left( p \right) < 0$), indicating that an immediate release is fairly unlikely but the likelihood of being released the next day increases over the already elapsed time spent in the hospital.

### Bayesian diagnostics

To assess the reliability of results, we examined the simulated MCMCs to see whether they converged and mixed (i.e., whether they reached a stationary process where all simulated values were drawn from the same a posteriori distribution used for parameter computation, credibility intervals and hypothesis tests).

First, we used effective sample sizes reported in Table 4, based on the total number of simulations and the extent of autocorrelation, to assess how informative our simulated MCMC parameters were (Table A5 in the Additional file 1: Appendix shows the average autocorrelation and Table A6 in the Additional file 1: Appendix reports the effective sample size for all MCMCs). For example, the effective sample size of 9,260 for open abdominal hernia repair indicated that when accounting for the autocorrelation in the corresponding Markov chain, the 100,000 simulations comprised as much information as would a sample of 9,260 independent and identically distributed (iid) observations. Although effective sample sizes were relatively small for some procedures, we considered effective sample sizes $> 1000$ to be sufficient to derive *a-posteriori* distributions. In addition, the acceptance rates of all procedures ranged from 0.3 to 0.4, which indicates a good performance of the Metropolis–Hastings-algorithm. Higher values would indicate too small steps in the search algorithm and bear the risk of omitting part of the potential parameter space, whereas too low acceptance rates would point towards too large steps in the search algorithm, which would indicate a random search pattern and the risk of, say, rushing too fast through the potential parameter space.

To assure convergence, we also examined various plots to graphically assess whether MCMCs were well behaved and whether convergence could be observed. First, MCMCs for all estimated parameters across all procedures yielded no visible trends or heteroscedasticities, suggesting converged (stationary) processes. Second, histograms of MCMC samples yielded unimodal parameter distributions, which again suggests a converged MCMC, and would be unlikely in cases of non-convergence. Third, the number of lags in autocorrelation functions suggested only a few statistically significant lags in MCMCs, again implying that they converged during the burn-in. Finally, dividing MCMCs in half and comparing kernel densities for simulated values yielded extremely similar patterns, which again indicates that mean, standard deviation and the shapes of the distributions did not significantly differ across the simulation process. This further supports the assertion that MCMCs converged during the burn-in process and followed a stationary process throughout the MCMC simulation process.

## Discussion

We estimated the impact of change in hospital payment from PDs to PRGs on LoS. We analyzed 14 general surgery, urology and gynecology procedures with mixed findings. Introduction of PRGs was intended, inter alia, to create incentives to reduce costs per case and thereby reduce LoS. Hospitals reduced LoS for half for procedures examined, mostly urological procedures. For those procedures, LoS declined by 11% to 20%. One potential explanation is that most urology procedures analyzed are more invasive; i.e., open procedures, which are characterized by long LoS, mainly due to lengthy recovery. In contrast, most of the general surgical procedures we examined were laparoscopic, and characterized by shorter LoS and faster recovery. It is more feasible to reduce LoS for invasive procedures by focusing on recovery, than laparoscopic procedures where the LoS are already short [30–34].

Inspecting the entire distributions of the length of stay before and after reform indicates that the reform had its main effect in the middle of the distributions around the mean and median of LoS, whereas the 95^th^ and 99^th^ percentile exhibit only minor changes. This supports our argument that the reform had the strongest impact on “average patients”, while decisions concerning more complicated or more severe cases may have been less subject to economic considerations.

Similar to European countries, one objective of introducing PRGs in Israel was to reduce waiting times [35]. By setting higher marginal prices for procedures with long waiting lists when replacing the previous PD payment scheme, the MoH expected hospitals to reduce LoS and thus to increase patient turnover [20] as observed in Norway [35, 36]. Indeed, hospital and ward directors, and surgeons reported that LoS decreased, and volumes of care increased after the PRG reform reducing waiting times, particularly in urology and orthopedics wards[37].

It is possible that the decrease in LoS released some resources to treat other patients, maybe reducing waiting times. Since the number of beds in Israel did not decrease [38], it is possible that shorter LoS allowed hospitals to increase productivity by performing additional procedures. Administrative data, however, enable only a rough assessment of whether this objective was achieved, in part, because actual waiting lists are not available.

### Unconventional methodology

For this study, we performed survival analyses to estimate relative changes in LoS. This methodology is particularly well-suited to these data and research questions. First, LoS are time-until-event data, for which survival analysis and underlying distribution assumptions were specifically designed. Second, measuring relative change in LoS in a context of payment reform allowed us to examine various patterns of LoS across procedures and patient groups. Patients with more comorbid conditions, for example, were likely to have longer LoS both before and after reform. We argue that similar relative changes by age, sex and comorbidity may be more likely than similar absolute changes, although we acknowledge that mixed effects linear regression models, for example, might yield comparable results. Third, since our observations were grouped by age, gender, procedure, hospital and year (rather than individual data), no variance was observed within those groups. A frequentist approach would underestimate the error variance and lead to biased results. The Bayesian approach allowed us to overcome the problem of lack of variance within the data groups because it is a simulation-based approach which compares many simulated outcomes with the observed real-world data, and does not require the computation of error covariance matrices.

Finally, we considered the Bayesian estimation to provide more relevant results for policymakers, since we present credibility intervals and cumulative distribution functions in addition to the usually reported point estimates for each procedure. In particular, Fig. 1 may enable policymakers to make more informed decisions based on the cumulative probabilities, rather than having to rely solely on a dichotomous distinction between significance and none-significance based conventional thresholds. This may be particularly beneficial if policymakers are not only interested in a yes or no decision. For example, policymakers will know whether a significant decrease of LoS was observed in a certain procedure. If the results were inconclusive for this procedure, they might also be interested in the probability of an increase of LoS for this procedure after the reform or in the probability of this procedure of not exceeding a certain threshold, e.g. a 10% increase. This may be informative for policymakers considering keeping the PRG payment also for the 7 out of 14 procedures where no significant decrease in LoS was observed. Both p-values and the probability of a time ratio < 1 reported in Table 4 represent tests only for one specific null hypothesis, whereas readers may alter the null hypothesis by moving on the *x*-axis and observe the resulting probabilities on the *y*-axis in Fig. 1.

### Implications for Israel

Our findings complement existing PRG reform research in Israel. Using a diff-in-diff analysis at the ward level, Waitzberg et al. [28] found no association between payment reform, change in LoS or patient turnover compared to wards where no new PRG codes were created, even when analyzing wards separately. We instead, examined effects of payment reform on specific procedures. There were significant reductions in LoS for half the procedures we examined, predominantly urological procedures. Thus, ward-level analyses may have been insufficiently sensitive; change for some procedures performed on patients on the same ward may have been negated by consistent LoS for other procedures.

In a qualitative study examining the effects of PRG reform, hospital managers and physicians reported that reimbursement for many PRGs were insufficient to cover costs, which tempered incentive for hospitals to increase patient turnover. The exception they reported was for orthopedic and urological procedures, for which LoS decreased and patient turnover increased, as found here. PRG-based payments alone do not incentivize patient turnover if prices do not allow for some marginal profit [39]. In fact, when comparing the PRG tariff with the average payment calculated as the PD tariff multiplied with the average LoS, we noticed that after the PRG reform, hospitals received on average payments twice as high (for a detailed list of the MoH tariffs and the difference after the reform see Table A7 in the Additional file 1: Appendix). The exception was Anti-reflux surgery and Open splenectomy, for which the payments were reduced by 20%. While the payments calculated are a rough estimation because hospitals receive in practice lower payments due to caps and discounts given to the HPs, we do not see a clear trend in time ratios of LoS decrease and the difference in payment.

It is widely acknowledged that shortening LoS through economic incentives may negatively affect the quality of care, especially in cases where patients are released too early. Israeli policymakers should therefore pay attention to the effects of decreases in LoS and assure that these decreases do not result in poorer health outcomes. Unfortunately, the MoH monitors indicators of process and quality of care at the hospital level, and only for some procedures, which do not include those analyzed in this study[29, 40]. If the payment reform undermined quality of care, shorter LoS and higher patient turnover might not be efficient or desirable.

### Implications for other countries

The finding that LoS did not significantly decline for half of the procedures examined (mostly general surgery and gynecology procedures) can be explained by various moderating factors which attenuated the effects of the PRG reform [37]. The first is the limited availability of healthcare resources in Israeli hospitals. In a stretched system where resources are limited, reducing LoS may be more difficult than in contexts with more resources and flexibility. Bed occupancy rates were the second highest, nurses per bed ratios are among the lowest and LoS was the third shortest among OECD countries even before the reform (curative beds occupancy rates were 93.3% in Israel compared to the OECD average of 75.2%). Acute care LoS in Israel was 5 days in the last decade, compared to the OECD average of 6.7 [41]. Second, the public nature of hospitals reduces the possibility to select patients or profitable procedures, which limited the potential to respond to the PRG reform [42]. Third, conflicting incentives such as retrospective (end-of-year) payments by the MoH to cover hospital deficits may limit responsiveness to payments’ incentives. Capped income and agreements between hospitals and HPs further suppress productivity incentives if costs are not recouped. Fourth, hospital managers and physicians reported that other considerations outweigh economic concerns such as commitment to patients and clinical needs; significant decreases in LoS would undermine quality of care [37]. Each of these factors should be considered when expanding PRG payments. PRG pricing should ensure that tariffs exceed costs, and that more resources be made available for hospitals to respond to and benefit from economic incentives.

These findings are applicable to other systems since these same moderating factors may impede payment reform to achieve desired objectives in other countries, or lead to unintended consequences. Nation-specific factors from other countries should be considered when implementing DRG-like payment programs.

Our findings are in line with previous research suggesting that DRG-like payment reform has limited and inconsistent effects on hospital activity; results vary depending on context and country [6]. For example, a systematic review found no clear trend in volume of care, and no consistent or systematic differences in mortality rates and quality of care, with considerable differences across countries and clinical domains [9]. In Germany, LoS and hospital revenues dropped but mixed results were found on patient turnover and other outcomes such as case severity and homogeneity of DRG groups [10]. In Norway, care volume increased for medical but not surgical patients [43] whereas in Italy, volume increased for surgical but not medical patients [44]. In England, care volume also increased but LoS decreased slightly [11, 12], whereas the opposite was observed in Central Asia, Central and Eastern Europe [8]. Introduction of DRG-like payments led to improved technical efficiency, i.e., hospitals reduced cost per case without harming quality of care or increasing productivity in Portugal [45], Finland [46] and Norway [36] while in Austria, technical efficiency did not improve [47]. Systematic reviews report mixed effects of DRGs on hospital activity [6, 9].

DRG payments have different effects in different countries and contexts. Simplistic evaluations of DRGs and facile comparisons between countries can lead to biased interpretations, inappropriate and adverse policy and political consequences [48]. New programs and changing payment policies indicate persisting needs for research and evaluation. Some countries such as France, Germany and Estonia experienced adverse and unintended consequences from DRGs, and in 2019 reduced the share of DRGs in relation to fixed payments (e.g., global budgets), others like Slovakia adopted DRGs that same year [49]. Investigating the direct and indirect impact of DRGs across contexts is fundamental for a successful design and implementation.

### Limitations

First, we did not have access to all costing data for procedures. Data on the actual costs of hospital care at the national level in Israel and other countries is limited, if they exist at all. Acquiring cost data from each hospital was beyond the scope of this study. This lack has two implications. First, the differences between pricing (payment tariff) and cost might be small, non-existent or negative, thus undermining incentives for change in admission and treatment policy. Therefore, our study cannot pinpoint the reason for the (lack of) changes in LoS. Second, we were unable to observe the exact reimbursements and the corresponding annual caps for the hospitals. We include the year-fixed effects to capture potential year-specific effects, and argue that annual changes in maximum reimbursements are adequately addressed by this methodology.

Second, our data were grouped by procedure and age-and-sex groups at the hospital level, not the patient-level; we did not have individual LoS data and could not assess LoS variance within groups. However, with the data grouped by age, sex, procedure, hospital and year, the uninflated dataset (i.e. before accounting for group sizes) already comprised 24,287 data points, which corresponded to 87,559 individual cases. There is a tradeoff between detailed, patient-level data from a few hospitals which might not be representative versus less detailed data from all hospitals. We opted for the latter to assess the impact of payment reform at the national level, and attempted to overcome this data limitation by Bayesian estimation.

Third, the data structure and the extreme outliers (up to the 26-fold of the value of the 95%-quantiles) led to problems with the estimation algorithms. The raw data indicates that the maximum stays and the 95% quantiles decreased to some extent after reform, but the main effect of the reform was observed around the mean and median of the distributions. Outliers were computed for each procedure over the full period. The fraction of observations considered as outliers varies around 5% over the years and shows no clear shift around the year of reform.

Fourth, our analyses were restricted to 14 procedures and inclusion was limited to procedures with 200+ cases per year to ensure that findings would not merely be a result of stochastic processes. Changes in LoS may have occurred for procedures not included in our analyses, in particular those for which reimbursements are still made per diem. While the 14 procedures might not provide a full picture of hospitals’ activities, by analyzing only procedures created in the second wave, the simultaneous introduction of the PRG payment allows a more reliable comparison between the various procedures.

Fifth, we excluded private hospitals, which represent 3% of the beds, but about 30% of the procedures [50]. These hospitals select simple cases and procedures, primarily as outpatient [42]. We assume that since one of our exclusion criteria was that procedures can be performed only in inpatient settings, the bias of not including private hospitals is low. One may speculate that if we had these cases in our analysis, the effects of the reform could have been more substatial, because it is easier to shorten LoS for simple cases, which have higher chances to have been performed in private hospitals.

Finally, the data included no information about the supplier characteristics. Reliable information about the numbers of surgeons, the number of hospital beds per ward or the actual waiting times were not available. We are not aware of noteworthy changes in the hospital infrastructure of or noteworthy changes in the demographic characteristics of the population, however, and argue that including years as random effects part in the fixed effects model would have covered such unobserved, year-specific information.

## Conclusions

Analyses of the effects of DRG-like payments can be more complex than first assumed. The effects of different payment mechanism may differ across different procedures. This depends on the grouping of patients, pre-reform LoS, the pricing, the technology (invasive or laparoscopic), demand elasticity and many other non-economic considerations such as physicians’ preferences, and commitment to the patient, to mention only a few. We found significant decreases in LoS for invasive procedures with longer recovery times whereas laparoscopic procedures with already short LoS showed no significant change; LoS cannot drop below a certain minimum. Conclusions as to payment reforms should thus be made with caution and consideration for varied effects across procedures, wards, and hospitals. Finally, context matters and country-specific factors can promote, moderate or impede responses to economic incentives. Aggregate and over-simplified data can bias findings and mislead policy makers.

## Supplementary Information

**Additional file 1**. Appendix.

## Data Availability

The data that support the findings of this study are available from the Israel Ministry of Health, but restrictions apply to the availability of these data, which were used under license for the current study, and so are not publicly available. Data are however available from the authors upon reasonable request and with permission of the Israel Ministry of Health.

## Footnotes

1 Estimating the models including the top 5% was attempted as a sensitivity analysis but reached no convergence, i.e. the models cannot be estimated if the 5% outliers are included.
