# Supplementary material for: It probably worked: a Bayesian approach to evaluating the introduction of activity-based hospital payment in Israel
Source: Isr J Health Policy Res. 2022 Feb 15;11:8. doi: 10.1186/s13584-022-00515-y (PMC8845384; doi:10.1186/s13584-022-00515-y)
Supplement: Supplementary file 1 — Additional file 1. Appendix. [file 13584_2022_515_MOESM1_ESM.docx]

# Appendices:

| table A1: ICD9 codes related to PRG codes | | | | | |
| --- | --- | --- | --- | --- | --- |
|  |  |  |  |  |  |
| PRG code | **Name of procedure** | **Main ICD9 code** |  | **In combination with codes from this column** |  |
| G0202 | Anterior abdominal wall hernia repair, excluding POVH, open | 53.69 | Other and open repair of other hernia of anterior abdominal wall with graft or prosthesis |  |  |
| G0203 | Anterior abdominal wall hernia repair, excluding POVH, laparoscopic | 53.59 | Repair of other hernia of anterior abdominal wall |  |  |
|  |  | 53.63 | Other laparoscopic repair of other hernia of anterior abdominal wall with graft or prosthesis |  |  |
| G0204 | Diaphragmatic hernia repair, laparoscopic | 53.71 | Laparoscopic repair of diaphragmatic hernia, abdominal approach |  |  |
|  |  | 53.83 | Laparoscopic repair of diaphragmatic hernia, with thoracic approach |  |  |
|  |  | 53.82 | Repair of parasternal hernia | 54.21 | Laparoscopy |
| G0208 | Esophagogastric sphincteric competence creation | 44.66 | Other procedures for creation of esophagogastric sphincteric competence |  |  |
|  |  | 44.67 | Laparoscopic procedures for creation of esophagogastric sphincteric competence |  |  |
| G0210 | Percutaneous nephrostomy, including fragmentation | 55.04 | Percutaneous nephrostomy with fragmentation |  |  |
| G0214 | Partial nephrectomy, open | 55.4 | Partial nephrectomy |  |  |
| G0215 | Partial nephrectomy, laparoscopic | 55.4 | Partial nephrectomy | 17.42 | Laparoscopic robotic assisted procedure |
|  |  |  |  | 54.21 | Laparoscopy |
| G0216 | Complete nephrectomy, including ureterectomy, open | 55.51 | Nephroureterectomy |  |  |
|  |  | 55.52 | Nephrectomy of remaining kidney |  |  |
|  |  | 55.54 | Bilateral nephrectomy |  |  |
| G0217 | Complete nephrectomy, including ureterectomy, laparoscopic | 55.51 | Nephroureterectomy | 17.42 | Laparoscopic robotic assisted procedure |
|  |  | 55.52 | Nephrectomy of remaining kidney | 54.21 | Laparoscopy |
|  |  | 55.54 | Bilateral nephrectomy |  |  |
| G0220 | Ureteroscopy, diagnostic, with/without biopsy | 56.31 | Ureteroscopy |  |  |
|  |  | 56.33 | Closed endoscopic biopsy of ureter |  |  |
| G0221 | Ureteroscopy, therapeutic, including RIRS | 56.31 | Ureteroscopy | 55.11 | Pyelotomy |
|  |  |  |  | 55.87 | Correction of ureteropelvic junction |
|  |  |  |  | 56.0 | Transurethral removal of obstruction from ureter and renal pelvis |
|  |  |  |  | 56.2 | Ureterotomy |
|  |  |  |  | 59.8 | Ureteral catheterization |
| G0230 | Salpingectomy, laparoscopic, unilateral/bilateral | 66.4 | Total unilateral salpingectomy | 54.21 | Laparoscopy |
|  |  | 66.51 | Removal of both fallopian tubes at same operative episode |  |  |
|  |  | 66.52 | Removal of remaining fallopian tube |  |  |
|  |  | 66.62 | Salpingectomy with removal of tubal pregnancy |  |  |
|  |  | 66.63 | Bilateral partial salpingectomy, not otherwise specified |  |  |
|  |  | 66.69 | Other partial salpingectomy |  |  |
| G0231 | Salpingo-oophorectomy, laparoscopic, unilateral/bilateral | 65.41 | Laparoscopic unilateral salpingo-oophorectomy |  |  |
|  |  | 65.63 | Laparoscopic removal of both ovaries and tubes at same operative episode |  |  |
|  |  | 65.64 | Laparoscopic removal of remaining ovary and tube |  |  |
| G0256 | Repair of corneal injury | 11.51 | Suture of corneal laceration |  |  |
|  |  | 11.53 | Repair of corneal laceration or wound with conjunctival flap |  |  |
| G0290 | Splenectomy, open, partial/complete | 41.43 | Partial splenectomy |  |  |
|  |  | 41.5 | Total splenectomy |  |  |

Appendix Table A2: Changes in patient characteristics after removal of outliers

|  | full sample | | | | after removal of outliers | | | |
| --- | --- | --- | --- | --- | --- | --- | --- | --- |
| Procedure | N | median age group | median Charlson index | percent women | N | median age group | percent women | median charlson index |
|  |  |  |  |  |  |  |  |  |
| **General surgery** |  |  |  |  |  |  |  |  |
| Open abdom. hernia rep. | 2674 | 55-64 | 0 | 54.08% | 2549 | 55-64 | 53.83% | 0 |
| Lap. abdom. hernia rep. | 5917 | 45-54 | 0 | 55.18% | 5628 | 45-54 | 54.74% | 0 |
| Lap. diaphragm. hernia rep. | 1312 | 55-64 | 0 | 68.37% | 1248 | 55-64 | 68.83% | 0 |
| Anti-reflux surgery | 2742 | 35-44 | 0 | 51.57% | 2608 | 35-44 | 51.92% | 0 |
| Open splenect. | 4685 | 55-64 | 1 | 47.13% | 4451 | 55-64 | 47.72% | 1 |
|  |  |  |  |  |  |  |  |  |
| **Urology** |  |  |  |  |  |  |  |  |
| Perct. nephrostomy | 5620 | 45-54 | 0 | 39.22% | 5361 | 45-54 | 38.76% | 0 |
| Open part. nephrect. | 3068 | 55-64 | 2 | 38.18% | 2921 | 55-64 | 38.36% | 2 |
| Lap. part. nephrect. | 1305 | 55-64 | 2 | 37.32% | 1245 | 55-64 | 37.67% | 2 |
| Open complete nephrect. | 7189 | 55-64 | 2 | 42.41% | 6832 | 55-64 | 42.75% | 2 |
| Lap. complete nephrect. | 1222 | 55-64 | 2 | 40.83% | 1161 | 55-64 | 41.43% | 2 |
| Diag. ureterosc. | 8538 | 55-64 | 0 | 30.93% | 8133 | 55-64 | 30.68% | 0 |
| Therap. ureterosc. | 22647 | 45-54 | 0 | 28.70% | 21517 | 45-54 | 28.31% | 0 |
|  |  |  |  |  |  |  |  |  |
| **Gynecology** |  |  |  |  |  |  |  |  |
| Lap. salpingect. | 5972 | 25-34 | 0 | 100.00% | 5680 | 25-34 | 100% | 0 |
| Lap. salp.-oophorect. | 14708 | 45-54 | 0 | 100.00% | 13984 | 45-54 | 100% | 0 |
| **Overall** | 87599 | 45-54 | 0 | 53.04% | 83318 | 45-54 | 52.94% | 0 |

Appendix Table A3: Effects of outlier removal on LoS data

|  | full sample including outliers | | | | | restricted sample excluding outliers | | | | |  |
| --- | --- | --- | --- | --- | --- | --- | --- | --- | --- | --- | --- |
| Procedure | N | mean | s.e. of mean | 95% quantile | maximum reported | N | mean | s.e. of mean | 95% quantile | maximum reported | N excluded |
|  |  |  |  |  |  |  |  |  |  |  |  |
| **General surgery** |  |  |  |  |  |  |  |  |  |  |  |
| Open abdom. hernia rep. | 2674 | 4.08 | 0.12 | 11 | 151 | 2549 | 3.02 | 0.04 | 7.5 | 11 | 125 |
| Lap. abdom. hernia rep. | 5917 | 3.80 | 0.10 | 10 | 266 | 5628 | 2.85 | 0.03 | 7.2 | 10 | 289 |
| Lap. diaphragm. hernia rep. | 1312 | 6.48 | 0.30 | 19 | 221 | 1248 | 4.80 | 0.09 | 12 | 19 | 64 |
| Anti-reflux surgery | 2742 | 18.83 | 0.62 | 76 | 545 | 2608 | 13.37 | 0.32 | 55.4 | 76 | 134 |
| Open splenect. | 4685 | 15.74 | 0.24 | 39.83 | 358 | 4451 | 13.22 | 0.13 | 31 | 39.83 | 234 |
|  |  |  |  |  |  |  |  |  |  |  |  |
| **Urology** |  |  |  |  |  |  |  |  |  |  |  |
| Perct. nephrostomy | 5620 | 6.64 | 0.05 | 12 | 94 | 5361 | 6.04 | 0.03 | 10.33 | 12 | 259 |
| Open part. nephrect. | 3068 | 7.72 | 0.12 | 15.25 | 185 | 2921 | 6.85 | 0.05 | 12 | 15.25 | 147 |
| Lap. part. nephrect. | 1305 | 6.31 | 0.08 | 10 | 60 | 1245 | 5.92 | 0.05 | 9 | 10 | 60 |
| Open complete nephrect. | 7189 | 9.77 | 0.12 | 22 | 406 | 6832 | 8.32 | 0.05 | 17 | 22 | 357 |
| Lap. complete nephrect. | 1222 | 7.53 | 0.15 | 15.33 | 73 | 1161 | 6.59 | 0.07 | 12 | 15.33 | 61 |
| Diag. ureterosc. | 8538 | 4.23 | 0.07 | 9 | 266.5 | 8133 | 3.55 | 0.02 | 7 | 9 | 405 |
| Therap. ureterosc. | 22647 | 3.88 | 0.02 | 7.75 | 277 | 21517 | 3.37 | 0.01 | 6.09 | 7.75 | 1130 |
|  |  |  |  |  |  |  |  |  |  |  |  |
| **Gynecology** |  |  |  |  |  |  |  |  |  |  |  |
| Lap. salpingect. | 5972 | 2.87 | 0.02 | 4.86 | 27 | 5680 | 2.67 | 0.01 | 3.88 | 4.86 | 292 |
| Lap. salp.-oophorect. | 14708 | 3.19 | 0.02 | 5.87 | 103 | 13984 | 2.94 | 0.01 | 5 | 5.87 | 724 |
| **Overall** | 87599 | 5.75 | 0.03 | 15.5 | 545 | 83318 | 4.86 | 0.02 | 12 | 76 | 4281 |

Table A4: Full estimation results

|  |  | general surgery | | | | | | | | | gynecology | | | |
| --- | --- | --- | --- | --- | --- | --- | --- | --- | --- | --- | --- | --- | --- | --- |
|  | Open abdom. hernia repair | | Lap. abdom. hernia repair | | Lap. diaphragm. hernia repair | | Anti-reflux surgery | | Open splenectomy | | Lap. salpingect. | | Lap. salp.-oophorect. | |
|  |  |  |  |  |  |  |  |  |  |  |  |  |  |  |
| post-reform | 0.867 | (0.718; 1.034) | 0.974 | (0.833; 1.118) | 1.017 | (0.865; 1.172) | 1.050 | (0.896; 1.216) | 0.894 | (0.784; 1.002) | 0.957 | (0.864; 1.055) | 0.929 | (0.840; 1.021) |
|  |  |  |  |  |  |  |  |  |  |  |  |  |  |  |
| Charlson index | 1.140 | (1.100; 1.181) | 1.115 | (1.085; 1.145) | 1.000 | (0.949; 1.056) | 1.434 | (1.360; 1.510) | 1.051 | (1.038; 1.065) | 0.882 | (0.839; 0.927) | 1.027 | (1.018; 1.037) |
| female | 1.160 | (1.107; 1.212) | 1.163 | (1.130; 1.197) | 1.050 | (0.989; 1.112) | 1.103 | (1.044; 1.165) | 0.817 | (0.789; 0.846) | n/a | | n/a | |
|  |  |  |  |  |  |  |  |  |  |  |  |  |  |  |
| Age | 1.001 | (0.995; 1.008) | 1.010 | (1.008; 1.012) | 0.963 | (0.959; 0.967) | 0.920 | (0.917; 0.924) | 1.001 | (0.998; 1.005) | 0.987 | (0.981; 0.992) | 0.998 | (0.996; 1.001) |
| age squared | 1.000 | (1.000; 1.000) | 1.000 | (1.000; 1.000) | 1.000 | (1.000; 1.000) | 1.001 | (1.001; 1.001) | 1.000 | (1.000; 1.000) | 1.000 | (1.000; 1.000) | 1.000 | (1.000; 1.000) |
|  |  |  |  |  |  |  |  |  |  |  |  |  |  |  |
| hospital in periphery | 1.086 | (1.031; 1.142) | 0.960 | (0.931; 0.991) | 0.714 | (0.668; 0.760) | 1.169 | (1.079; 1.259) | 1.032 | (0.986; 1.078) | 1.085 | (1.069; 1.100) | 1.145 | (1.131; 1.159) |
| tertiary care hospital | 1.207 | (1.147; 1.270) | 0.989 | (0.959; 1.019) | 0.828 | (0.783; 0.875) | 1.149 | (1.084; 1.214) | 1.213 | (1.168; 1.259) | 1.080 | (1.064; 1.095) | 1.014 | (1.003; 1.026) |
|  |  |  |  |  |  |  |  |  |  |  |  |  |  |  |
| Hospital owned by |  |  |  |  |  |  |  |  |  |  |  |  |  |  |
| NGO | 0.969 | (0.907; 1.032) | 0.738 | (0.706; 0.769) | 0.893 | (0.820; 0.969) | 0.908 | (0.842; 0.975) | 0.849 | (0.806; 0.891) | 0.902 | (0.885; 0.920) | 0.726 | (0.715; 0.738) |
| Health plan | 0.834 | (0.788; 0.881) | 0.838 | (0.809; 0.868) | 0.803 | (0.756; 0.851) | 0.945 | (0.886; 1.006) | 0.987 | (0.947; 1.028) | 0.815 | (0.801; 0.829) | 0.781 | (0.772; 0.790) |
|  |  |  |  |  |  |  |  |  |  |  |  |  |  |  |
| constant | 2.143 | (1.759; 2.565) | 1.733 | (1.576; 1.886) | 12.875 | (11.086; 14.658) | 33.906 | (30.400; 37.449) | 12.850 | (11.544; 14.179) | 3.359 | (3.011; 3.714) | 3.348 | (3.064; 3.635) |
| ln(p) | 0.566 | (0.537; 0.594) | 0.622 | (0.604; 0.640) | 0.768 | (0.729; 0.806) | 0.379 | (0.352; 0.406) | 0.530 | (0.508; 0.553) | 1.451 | (1.431; 1.470) | 1.233 | (1.220; 1.245) |
|  |  |  |  |  |  |  |  |  |  |  |  |  |  |  |
| random part |  |  |  |  |  |  |  |  |  |  |  |  |  |  |
| var($u_{i}$) | 0.017 | (0.004; 0.037) | 0.012 | (0.003; 0.025) | 0.010 | (0.001; 0.024) | 0.011 | (0.002; 0.025) | 0.008 | (0.002; 0.016) | 0.006 | (0.002; 0.012) | 0.006 | (0.002; 0.012) |

Table A4 Full estimation results (continued)

|  | urology | | | | | | | | | | | | | |
| --- | --- | --- | --- | --- | --- | --- | --- | --- | --- | --- | --- | --- | --- | --- |
|  | Perct. nephrostomy | | Open part. nephrect. | | Lap. part. nephrect. | | Open complete nephrect. | | Lap. complete nephrect. | | Diag. ureterosc. | | Therap. ureterosc. | |
|  |  |  |  |  |  |  |  |  |  |  |  |  |  |  |
| post-reform | 0.853 | (0.769; 0.940) | 0.871 | (0.756; 0.990) | 0.801 | (0.640; 0.967) | 0.891 | (0.767; 1.013) | 0.950 | (0.843; 1.061) | 0.852 | (0.759; 0.949) | 0.892 | (0.783; 1.002) |
|  |  |  |  |  |  |  |  |  |  |  |  |  |  |  |
| Charlson index | 1.074 | (1.057; 1.092) | 1.026 | (1.012; 1.039) | 0.995 | (0.977; 1.011) | 1.048 | (1.038; 1.059) | 1.059 | (1.038; 1.080) | 1.026 | (1.013; 1.038) | 1.051 | (1.039; 1.063) |
| female | 1.054 | (1.036; 1.073) | 1.003 | (0.978; 1.028) | 0.948 | (0.923; 0.974) | 0.967 | (0.946; 0.988) | 0.983 | (0.942; 1.023) | 1.037 | (1.017; 1.058) | 1.061 | (1.050; 1.072) |
|  |  |  |  |  |  |  |  |  |  |  |  |  |  |  |
| age | 0.997 | (0.994; 0.999) | 0.991 | (0.988; 0.993) | 0.998 | (0.994; 1.003) | 0.995 | (0.993; 0.997) | 0.998 | (0.994; 1.002) | 0.998 | (0.996; 1.001) | 0.990 | (0.988; 0.991) |
| age squared | 1.000 | (1.000; 1.000) | 1.000 | (1.000; 1.000) | 1.000 | (1.000; 1.000) | 1.000 | (1.000; 1.000) | 1.000 | (1.000; 1.000) | 1.000 | (1.000; 1.000) | 1.000 | (1.000; 1.000) |
|  |  |  |  |  |  |  |  |  |  |  |  |  |  |  |
| hospital in periphery | 0.871 | (0.849; 0.892) | 1.084 | (1.046; 1.122) | 1.022 | (0.970; 1.076) | 0.972 | (0.943; 1.000) | 0.969 | (0.880; 1.063) | 0.999 | (0.978; 1.021) | 1.152 | (1.139; 1.166) |
| tertiary care hospital | 1.058 | (1.037; 1.078) | 1.040 | (1.013; 1.066) | 1.044 | (1.011; 1.076) | 1.104 | (1.080; 1.128) | 1.111 | (1.062; 1.162) | 1.017 | (0.995; 1.040) | 1.005 | (0.994; 1.016) |
|  |  |  |  |  |  |  |  |  |  |  |  |  |  |  |
| Hospital owned by |  |  |  |  |  |  |  |  |  |  |  |  |  |  |
| NGO | 0.985 | (0.956; 1.015) | 1.023 | (0.983; 1.062) | 0.964 | (0.895; 1.036) | 0.757 | (0.736; 0.779) | 1.057 | (0.996; 1.122) | 0.913 | (0.888; 0.939) | 0.988 | (0.972; 1.005) |
| Health plan | 1.051 | (1.029; 1.073) | 0.906 | (0.881; 0.931) | 0.909 | (0.875; 0.943) | 0.901 | (0.879; 0.923) | 1.134 | (1.049; 1.222) | 1.062 | (1.039; 1.086) | 0.855 | (0.845; 0.864) |
|  |  |  |  |  |  |  |  |  |  |  |  |  |  |  |
| constant | 7.025 | (6.467; 7.603) | 8.205 | (7.401; 9.033) | 7.046 | (5.993; 8.158) | 9.802 | (8.985; 10.615) | 5.373 | (4.767; 5.989) | 3.590 | (3.277; 3.905) | 4.344 | (4.042; 4.659) |
| ln(p) | 1.150 | (1.130; 1.171) | 1.118 | (1.092; 1.145) | 1.491 | (1.448; 1.535) | 0.832 | (0.815; 0.849) | 1.086 | (1.043; 1.128) | 0.880 | (0.864; 0.897) | 1.052 | (1.042; 1.062) |
|  |  |  |  |  |  |  |  |  |  |  |  |  |  |  |
| random part |  |  |  |  |  |  |  |  |  |  |  |  |  |  |
| var($u_{i}$) | 0.006 | (0.002; 0.012) | 0.010 | (0.003; 0.021) | 0.023 | (0.007; 0.049) | 0.011 | (0.003; 0.023) | 0.006 | (0.002; 0.014) | 0.007 | (0.002; 0.015) | 0.008 | (0.002; 0.017) |

Table A5 Average autocorrelation in MCMCs

|  | General surgery | | | | | Gynecology | |
| --- | --- | --- | --- | --- | --- | --- | --- |
|  | Open abdom. hernia rep. | Lap. abdom. hernia rep. | Lap. diaphragm. hernia rep. | Anti-reflux surgery | Open splenect. | Lap. salpingect. | Lap. salp.-oophorect. |
| post-reform | 10.8 | 8.36 | 4.32 | 2.8 | 3.99 | 24.5 | 36.04 |
| Charlson index | 1.09 | 1.11 | 1.03 | 1.02 | 1.31 | 1.74 | 5.99 |
| female | 1.09 | 1.33 | 1.19 | 1.14 | 1.09 | n/a | n/a |
| age | 28.29 | 6.1 | 6.4 | 2.1 | 16.11 | 81.86 | 70.31 |
| age squared | 23.41 | 4.17 | 5.65 | 1.68 | 13.19 | 75.77 | 65.43 |
| hospital in periphery | 3.4 | 1.83 | 2.27 | 1.15 | 1.23 | 3.21 | 2.23 |
| tertiary care hospital | 1.2 | 1.95 | 1.53 | 1.56 | 2.27 | 1.69 | 1.45 |
|  |  |  |  |  |  |  |  |
| Hospital owned by |  |  |  |  |  |  |  |
| NGO | 5.22 | 2.54 | 2.2 | 1.58 | 1.6 | 5.36 | 1.78 |
| Health plan | 4.34 | 4.14 | 2.59 | 1.87 | 2.79 | 3.04 | 1.94 |
|  |  |  |  |  |  |  |  |
| constant | 36.8 | 17.54 | 6.19 | 6.2 | 23.08 | 89.53 | 74.58 |
|  |  |  |  |  |  |  |  |
| ln(p) | 1 | 1 | 1.03 | 1 | 1 | 1.02 | 1.02 |
|  |  |  |  |  |  |  |  |
| variance of random effect |  |  |  |  |  |  |  |
| year | 2.06 | 2.82 | 3.89 | 1.58 | 1.55 | 5.15 | 4.1 |

Table A5 Average autocorrelation in MCMCs (continued)

|  | Urology | | | | | | |
| --- | --- | --- | --- | --- | --- | --- | --- |
|  | Perct. nephrostomy | Open part. nephrect. | Lap. part. nephrect. | Open complete nephrect. | Lap. complete nephrect. | Diag. ureterosc. | Therap. ureterosc. |
| post-reform | 12.42 | 9.7 | 30.85 | 10.86 | 4.16 | 12.12 | 67.17 |
| Charlson index | 1.14 | 2.04 | 2.89 | 1.85 | 1.72 | 1.52 | 2.73 |
| female | 1.02 | 1.28 | 1.12 | 1.06 | 1.1 | 1 | 1 |
| age | 48.93 | 19.93 | 49.19 | 4.36 | 9.67 | 22.17 | 19.28 |
| age squared | 39.23 | 15.82 | 40.96 | 2.93 | 8.01 | 19.09 | 16.75 |
| hospital in periphery | 13.8 | 1.95 | 2.55 | 1.47 | 3.7 | 1.52 | 1.87 |
| tertiary care hospital | 2.98 | 2.54 | 1.47 | 1.12 | 2.78 | 1.76 | 5.94 |
|  |  |  |  |  |  |  |  |
| Hospital owned by |  |  |  |  |  |  |  |
| NGO | 1.83 | 2.44 | 11.53 | 2.75 | 3.24 | 2.72 | 14.65 |
| Health plan | 2.58 | 3.3 | 1.65 | 1.82 | 1.28 | 2.23 | 8.59 |
|  |  |  |  |  |  |  |  |
| constant | 59.69 | 26.11 | 60.35 | 13.5 | 9.53 | 23.75 | 50.37 |
|  |  |  |  |  |  |  |  |
| ln(p) | 1 | 1 | 1 | 1 | 1 | 1 | 1 |
|  |  |  |  |  |  |  |  |
| variance of random effect |  |  |  |  |  |  |  |
| year | 3.55 | 2.18 | 4.52 | 2.61 | 1.43 | 2.46 | 7.18 |

Table A6 Effective sample size of MCMCs

|  | General surgery | | | | | Gynecology | |
| --- | --- | --- | --- | --- | --- | --- | --- |
|  | Open abdom. hernia rep. | Lap. abdom. hernia rep. | Lap. diaphragm. hernia rep. | Anti-reflux surgery | Open splenect. | Lap. salpingect. | Lap. salp.-oophorect. |
| post-reform | 9260.31 | 11966.13 | 23133.84 | 35751.56 | 25060.27 | 4081.86 | 2774.33 |
| Charlson index | 92107.14 | 89829.48 | 96767.1 | 97749.36 | 76585.53 | 57372.86 | 16694.92 |
| female | 91778.21 | 75458.39 | 83708.58 | 87516.6 | 91885.09 | n/a | n/a |
| age | 3534.26 | 16399.6 | 15631.16 | 47614.5 | 6205.7 | 1221.6 | 1422.31 |
| age squared | 4272.27 | 23972.6 | 17713.43 | 59660.89 | 7578.66 | 1319.82 | 1528.26 |
| hospital in periphery | 29381.15 | 54601.87 | 44125.16 | 86713.19 | 81360.8 | 31193.45 | 44906.49 |
| tertiary care hospital | 83658.2 | 51343.86 | 65233.69 | 64156.8 | 43962.91 | 59067.89 | 68770.23 |
|  |  |  |  |  |  |  |  |
| Hospital owned by |  |  |  |  |  |  |  |
| NGO | 19165.15 | 39428.05 | 45468.24 | 63282.71 | 62543.04 | 18670.89 | 56145.16 |
| Health plan | 23029.09 | 24145.19 | 38660.85 | 53537.3 | 35836.58 | 32843.12 | 51629.45 |
|  |  |  |  |  |  |  |  |
| constant | 2717.3 | 5700.48 | 16143.3 | 16131.86 | 4332.55 | 1116.94 | 1340.91 |
|  |  |  |  |  |  |  |  |
| ln(p) | 100000 | 100000 | 97541.63 | 100000 | 100000 | 97648.55 | 97779.16 |
|  |  |  |  |  |  |  |  |
| variance of random effect |  |  |  |  |  |  |  |
| year | 48607.23 | 35455.54 | 25688.49 | 63170.95 | 64662.8 | 19403.11 | 24386.86 |

Table A6 Effective sample size of MCMCs (continued)

|  | Urology | | | | | | |
| --- | --- | --- | --- | --- | --- | --- | --- |
|  | Perct. nephrostomy | Open part. nephrect. | Lap. part. nephrect. | Open complete nephrect. | Lap. complete nephrect. | Diag. ureterosc. | Therap. ureterosc. |
| post-reform | 8050.26 | 10307.65 | 3241.02 | 9207.45 | 24059.07 | 8251.53 | 1488.81 |
| Charlson index | 87646 | 48959.24 | 34564.08 | 54194 | 58100.74 | 65848.49 | 36670.52 |
| female | 97584.17 | 77961.78 | 88923.77 | 94156.92 | 91237.95 | 100000 | 100000 |
| age | 2043.54 | 5017.15 | 2033.01 | 22929.28 | 10340.44 | 4510.28 | 5186.37 |
| age squared | 2549.14 | 6319.98 | 2441.61 | 34158.11 | 12477.21 | 5239.61 | 5970.38 |
| hospital in periphery | 7246.22 | 51339.03 | 39200.74 | 68258.93 | 27006.99 | 65664.88 | 53383.47 |
| tertiary care hospital | 33604.34 | 39325.54 | 68037.97 | 89149.44 | 35916.95 | 56772.03 | 16825.9 |
|  |  |  |  |  |  |  |  |
| Hospital owned by |  |  |  |  |  |  |  |
| NGO | 54544.76 | 41025.23 | 8671.8 | 36344.8 | 30878.09 | 36743.08 | 6827.52 |
| Health plan | 38718.21 | 30305.23 | 60657.24 | 54961.83 | 78297.2 | 44837.32 | 11643.89 |
|  |  |  |  |  |  |  |  |
| constant | 1675.33 | 3829.71 | 1656.93 | 7405.44 | 10496.69 | 4210.95 | 1985.4 |
|  |  |  |  |  |  |  |  |
| ln(p) | 100000 | 100000 | 100000 | 100000 | 100000 | 100000 | 100000 |
|  |  |  |  |  |  |  |  |
| variance of random effect |  |  |  |  |  |  |  |
| year | 28201.09 | 45899.96 | 22133.31 | 38302.12 | 70117.73 | 40632.48 | 13923.44 |

Table A7: PRG codes tariffs and average difference with PD payments calculated as a function of average length of stay (ALoS)

| **Procedure number** | **Abbreviated name of procedure** | **PRG code** | **Full name of procedure** | **MoH PRG tariff on 8.10.2014 in NIS** | **ALoS before reform in days (2005-2013)** | **ALoS up to day 3 (basic tariff)** | **ALoS from day 4 and on (reduced tariff)** | **payment before the reform (PD tariff * ALoS)** | **payment difference after the reform** | **Time ratio** |
| --- | --- | --- | --- | --- | --- | --- | --- | --- | --- | --- |
| 1 | Open abdom. hernia rep. | G0202 | Anterior abdominal wall hernia repair, excluding POVH, open | 19,746 | 3.167 | 3 | 0.167 | 8,807 | 10,939 | 0.867 |
| 2 | Lap. abdom. hernia rep. | G0203 | Anterior abdominal wall hernia repair, excluding POVH, laparoscopic | 19,746 | 2.884 | 3 |  | 8,075 | 11,671 | 0.974 |
| 3 | Lap. diaphragm. hernia rep. | G0204 | Diaphragmatic hernia repair, laparoscopic | 25,756 | 4.937 | 3 | 1.937 | 13,117 | 12,639 | 1.017 |
| 4 | Anti-reflux surgery | G0208 | Esophagogastric sphincteric competence creation | 28,480 | 13.463 | 3 | 10.463 | 33,877 | -5,397 | 1.050 |
| 5 | Open splenect. | G0290 | Splenectomy, open, partial/complete | 27,054 | 13.597 | 3 | 10.597 | 34,204 | -7,150 | 0.894* |
| 6 | Perct. nephrostomy | G0210 | Percutaneous nephrostomy, including fragmentation | 36,475 | 6.344 | 3 | 3.344 | 16,543 | 19,932 | 0.853** |
| 7 | Open part. nephrect. | G0214 | Partial nephrectomy, open | 34,233 | 7.143 | 3 | 4.143 | 18,488 | 15,745 | 0.871* |
| 8 | Lap. part. nephrect. | G0215 | Partial nephrectomy, laparoscopic | 34,233 | 6.333 | 3 | 3.333 | 16,516 | 17,717 | 0.801* |
| 9 | Open complete nephrect. | G0216 | Complete nephrectomy, including ureterectomy, open | 36,245 | 8.647 | 3 | 5.647 | 22,150 | 14,095 | 0.891* |
| 10 | Lap. complete nephrect. | G0217 | Complete nephrectomy, including ureterectomy, laparoscopic | 36,245 | 6.712 | 3 | 3.712 | 17,439 | 18,806 | 0.950 |
| 11 | Diag. ureterosc. | G0220 | Ureteroscopy, diagnostic, with/without biopsy | 11,611 | 3.764 | 3 | 0.764 | 10,260 | 1,351 | 0.852** |
| 12 | Therap. ureterosc. | G0221 | Ureteroscopy, therapeutic, including RIRS | 17,829 | 3.493 | 3 | 0.493 | 9,600 | 8,229 | 0.892* |
| 13 | Lap. salpingect. | G0230 | Salpingectomy, laparoscopic, unilateral/bilateral | 13,730 | 2.650 | 3 |  | 7,420 | 6,310 | 0.957 |
| 14 | Lap. salp.-oophorect. | G0231 | Salpingo-oophorectomy, laparoscopic, unilateral/bilateral | 13,730 | 2.964 | 3 |  | 8,299 | 5,431 | 0.929 |

**Notes:**

1. PD – per diem; PRG – procedure related group payment; MoH – Ministry of Health; ALoS – average length of stay
2. Basic PD tariff (up to day 3) in 2014 (payment code G00H2): NIS 2,800
3. PD tariff - reduced from day 4 and on in 2014 (payment code G00H4): NIS 2,435
4. Source of payment tariffs: MoH price list from the October, 8 2014. Available at: <https://www.health.gov.il/Subjects/Finance/Taarifon/Documents/081014abc.xlsx> [retrieved on November 16, 2021]
